# The non-selective Antarctic filter feeder *Salpa thompsoni* as a bioindicator of mercury origin

**DOI:** 10.1038/s41598-024-52770-5

**Published:** 2024-01-26

**Authors:** Adriana Wojdasiewicz, Anna Panasiuk, Magdalena Bełdowska

**Affiliations:** 1 Department of Chemical Oceanography and Marine Geology, Laboratory of Toxic Substances Transformation, Faculty of Oceanography and Geography, University of Gdańsk, Al. Marszałka Piłsudskiego 46, 81-378 Gdynia, Poland; 2 Department of Marine Biology and Biotechnology, Laboratory of Plankton Biology, Faculty of Oceanography and Geography, University of Gdańsk, Al. Marszałka Piłsudskiego 46, 81-378 Gdynia, Poland

**Keywords:** Environmental sciences, Ocean sciences, Marine biology, Marine chemistry

## Abstract

Hg is considered as the most toxic metal in the environment. Sources of Hg in the environment include burning fossil fuels, burning waste, and forest fires. The long residence time of the gaseous form in the atmosphere allows mercury to be transported over long distances. The pelagic tunicate *Salpa thompsoni* is an important component of the Antarctic environment. Over the past few decades an expansion of this species to the higher latitudes has been noted, mainly due to the ongoing climate change. The study material consisted of samples of *S. thompsoni* individuals, collected in the waters surrounding Elephant Island (Western Antarctic). Total mercury and five of its fractions were determined. Whole organisms were analyzed as well as internal organs: stomachs, muscle strips, and tunics. Obtained results showed that the highest concentrations of mercury in salps were observed in stomachs. With the Hg fraction results, it can be concluded that the main route of exposure of *S. thompsoni* to Hg is presumably absorption from the food—filtered organic and non-organic particles. Moreover, the process of transformation of simple soluble forms into organic forms of Hg in stomachs and intestines and its distribution to other tissues was observed.

## Introduction

Mercury (Hg) is a toxic metal introduced into the environment both from natural sources such as volcanic activity, fires, soil, rock erosion, and evaporation from water, land, and from anthropogenic sources, mainly the burning of fossil fuels, gold mining or waste ^1^. This chemical element methylates, bioaccumulates, and biomagnifies through the trophic chain, causing the highest concentrations in species at the highest levels of the food chain ^2^. Hg is a neurotoxin and in high concentrations causes irreversible changes in the nervous, immune, and reproductive systems and can also lead to death ^3,4^. Hg in the environment occurs in various forms: metallic, elemental Hg; inorganic Hg salts and organic Hg compounds which is the most bioavailable to the aquatic organisms ^5^. In the atmosphere, the main form of mercury is gaseous (Hg (0)), and its residence time is up to 18 months ^6^. During this time, air masses travel long distances due to the global atmospheric circulation and transport pollutants, mainly from industry, to regions distant from anthropogenic sources, including the Antarctic and Arctic areas ^7^. Hg is then deposited by wet and dry deposition on water and ice surfaces. In water, Hg is methylated, accelerating its incorporation into the trophic web ^8^. In the Southern Ocean there are other sources of trace metals, including upwelling processes, volcanic eruptions (also underwater), human activities (e.g., research stations), melting glaciers, and animal feces ^9^. Increasing concentrations of heavy metals in polar regions have been observed for many years ^10^, and this process has the potential to disrupt sensitive polar environments, including the Antarctic ecosystem.

*Salpa thompsoni* (Tunicata) is a species of pelagic tunicates that is abundant in the waters surrounding the Antarctic continent ^11–13^. However, in the last few decades, presumably as a result of climate change, there has been observed an expansion of salps to higher latitudes, simultaneously with their increasing abundance ^13^, which may have negative consequences for the whole Antarctic ecosystem, including the stability of the population of Antarctic krill (*Euphausia superba*), which is the dominant food for Antarctic predators ^14,15^. Representatives of *S. thompsoni* belong to the gelatinous plankton species and are made up of approximately 95% water ^16^. They are active but non-selective filter feeders and are able to feed on particles of a wide size range (1–1000 μm) ^17^. Salps reproduce sexually and asexually, and they are able to form dense blooms under favorable environmental conditions (temperature, sea ice cover size, food availability) ^18^. Moreover, these pelagic animals produce many fecal pellets rich in carbon and nitrogen, which sink to the ocean floor at a rapid rate ^17^. When forming dense blooms, they have a major role in the biogeochemical cycling of carbon ^19^.

Despite the increasing ecological importance of salps in the Antarctic region, there is still very little data on the content of toxic elements in their tissues. Results of the research which have been published so far by other authors are very rare and usually concern a larger group of zooplankton species and did not focus on the Antarctic salps e.g.^20^, and much more data relates to the Antarctic krill *E. superba* e.g.^21–23^. Taking the above facts into account the aim of this study was to assess the role of *S. thompsoni* as a vector of toxic Hg in the Antarctic environment. Additionally, to assess the transfer of this element in salp organisms, Hg fractions in whole organisms, in stomachs and intestines, in muscle belts and in whole bodies of tunicates were examined. The conducted research, as well as the results presented in this study, are among the first such detailed analyses of Hg presence in the Antarctic salps.

## Results and discussion

The mean THg concentration recorded in *S. thompsoni* individuals (n = 775) collected near Elephant Island in 2018 was 30.1 ng/g dw (dry weight), and the THg concentrations ranged from 11.8 ng/g dw to 63.9 ng/g dw (Table 1). These results were very similar to those obtained in 2007/2008 in the waters around South Georgia (mean 30 ng/g dw) by Seco et al. ^21^ and about 20% higher than in salps collected in 1999 near the Kerguelen Islands by Cipro et al. ^24^. Salps for this study were collected from the stations which were located close to the island, therefore increased values of THg obtained during this study may be related to penguin colonies that are located on Elephant Island. This island is considered one of the hot spots for the chinstrap penguins, however there are also colonies of macaroni and Adélie penguins ^25^. Mean concentrations of THg in feathers of chinstrap penguins can be up 600 ng/g dw ^26^, and in guano can be up to 1600 ng/g dw ^27^. Considering that salps are very effective continuous filter feeders ^17^, recorded concentrations may reflect the quality of organic molecules in the surrounding environment, which are a source of food for these representatives of zooplankton, however additional analyzes of particles in the water column would be necessary. Cipro et al. ^24^, who were mentioned above, collected samples for their research 20 years earlier in comparison to the presented studies. It is currently known that global climate change and associated cryosphere degradation has been observed for several decades in northern and southern hemispheres ^28,29^. Especially, close to the shores in the shelf areas there can be also additional sources of Hg from melting glaciers from which pollutants can be released ^30^. Continued decreasing ice cover, and thawing permafrost are leading to the re-emission of accumulated heavy metals that have been deposited there for decades. Consequently, there can be recorded an increase in the concentrations of them in the polar regions, and as a result of their loading into trophic webs ^31^.

**Table 1:** Basic descriptive statistics of total and fractional mercury concentrations [ng/g dw] in whole organisms, stomachs and intestines, and muscle strips and tunics of *Salpa thompsoni*, in 2018.

	THg	Hg_F1_	Hg_F2_	Hg_F3_	Hg_F4_	Hg_F5_
Whole organisms						
n	775		775			775
mean	30.1	4.8	19.4	0.5	5.3	0.1
median	29.6	4.5	19.6	0.5	4.9	0.1
min	11.8	0.9	10.1	0.3	0.4	0.0
max	63.9	15.0	36.4	0.7	11.7	0.1
SD	16.0	4.6	7.9	0.1	3.5	0.0
Stomachs and intestines						
n	777		777			777
mean	46.1	8.3	32.6	0.7	4.4	0.1
median	43.4	7.6	31.4	0.6	3.7	0.1
min	14.8	2.0	12.3	0.2	0.2	0.0
max	92.9	18.4	59.2	1.2	13.9	0.2
SD	19.6	4.9	9.4	0.2	5.0	0.1
Muscle strips and tunics						
n	777		777			777
mean	17.9	3.7	11.4	0.3	2.3	0.1
median	12.9	1.7	9.4	0.2	1.6	0.0
min	3.1	0.2	2.3	0.0	0.7	0.0
max	44.2	12.9	25.6	1.1	4.2	0.4
SD	12.6	4.0	6.9	0.4	1.2	0.1

Most studies of Hg concentrations in the Antarctic zooplankton usually focus on the Antarctic krill e.g.^21–23^. In case of *E. superba*, the results obtained by different authors vary greatly in different parts of the Antarctic. However, it can be considered that the results obtained for salps during these studies are in the upper range of values recorded for *E. superba* in various regions. For example, during surveys in the same region and at the same time as the current study the recorded mean range of THg concentrations varied from 17.9 ng/g dw (females) to 34.5 ng/g dw (juveniles) ^23^. In the WAP (Western Antarctic Peninsula) region between 2011 and 2015 the recorded range was between 4 ng/g dw and 19.4 ng/g dw ^22^. Higher values of the THg concentrations, from 6 ng/g dw to as high as 77 ng/g dw were noted by Seco et al. ^21^ in the Scotia Sea and Bargagli et al. ^30^ in the Ross Sea regions. Highly variable concentrations of THg in the *E. superba* tissues obtained by several authors may be related to the unique ecological characteristics of krill, but also physiological characteristics such as dense lipid content, a wide omnivorous diet ^32^, and their widespread occurrence ^20,33^. However, the results obtained for salps and krill are lower than those recorded for predatory representatives of zooplankton or animals at higher levels of the food chain. For comparison, a predatory Antarctic crustacean *Themisto gaudichaudii*, collected in late 2016 and early 2017 in the Scotia Sea, had an average THg concentration of 60 ng/g dw, a plankton-eating fish *Electrona antarctica* had 120 ng/g dw, and the Patagonian toothfish *Dissostichus eleginoides*, a predatory fish that can reach up to 2 m in length, had 200 ng/g dw ^20^. THg concentration in the grey-headed albatross (*Thalassarche chrysostoma*), a large predatory seabird, was 1430 ng/g dw ^20^, and in the Antarctic fur seals (*Arctocephalus gazella*) was recorded 6300 ng/g dw—from the Antarctic Peninsula region ^34^. In the Southern Ocean food web, the highest THg values have been noted in predatory species from the highest trophic levels (mammals, fish, birds) ^20,30,35^. Such results demonstrate the biomagnification process in the Antarctic trophic chain ^20^. Salps are almost at the beginning of the trophic chain, therefore the Hg concentrations recorded during these studies can be considered high.

### THg and Hg fractions in different body parts of *Salpa thompsoni*

Given the body parts of salps, a differential distribution of Hg was found between tissues (Table 1). Mean total Hg concentrations were more than twice as high in stomachs and intestines (46.1 ng/g dw) than in muscle strips and whole tunicates (17.9 ng/g dw) (Table 1). Increased concentrations of the metal in stomachs, intestines are presumably associated with the introduction of Hg into the body with a food. Similar results were obtained in the THg study in squids from the Atlantic Ocean, where total Hg concentrations in stomachs were 40% higher than in mantles ^36^. The preferred food for salps is phytoplankton, but because they are non-selective filter feeders, they can feed on particles with sizes ranging from 1 µm to even 1000 µm ^17^. The presented relationship of Hg concentrations in salps’ parts was opposite to that reported in predatory fish or cephalopods, where the highest Hg concentrations were found in muscle tissues ^37–39^. Similar to total Hg, its fractions: Hg_F1_, Hg_F2_, Hg_F3_ and Hg_F5_ showed during this study the highest values in stomachs and intestines (all fractions are described in the materials and methods section) (Table 1). The Hg_F4_ fraction reached the highest concentration in whole organisms, and this group included compounds such as HgO, HgSO_4_, HgF_2_. The increase in total Hg concentration in whole organisms was mainly related to bioaccumulation of a labile fraction 1 (mainly in combination with halogens) and a labile fraction 2 (organic forms and Hg bound to organic matter) (r = 0.88 and 0.93; *p* < 0.05, respectively) (Table 2). The same relationships were found in muscle girdles and tunics of salps (r = 0.89 and 0.98; *p* < 0.05). The Hg_F2_ fraction is easily biomagnified ^40^, which may facilitate Hg accumulation in salps from the food. A positive correlation was also recorded in muscle strips and tunicates between Hg_F3_ and Hg_F5_ concentrations (r = 0.96, *p* < 0.05) and in stomachs and intestines of *S. thompsoni* individuals (r = 0.71, *p* < 0.05) (Table 2). These fractions are the most stable, least toxic, non-reactive and their occurrence can be related to the common source of stable fractions, which is the mineral suspension ^41^. It is carried by meltwater, which probably indicates the incorporation of Hg into the trophic chain from melting glaciers ^42^. As was mentioned above, salps are not selective filter feeders, which gives a high probability of accumulating of this type of suspension. For example, Pakhomov et al. ^43^ even described cases of clogging of salp filtration systems as a result of high concentrations of inorganic matter in the Potter Cove (King George Island). The negative correlation of Hg_F4_ with fractions Hg_F1_ and Hg_F2_ in stomachs and intestines that was observed (r = − 0.74, *p* < 0.05) (Table 2) may indicate transformations of Hg from simple soluble forms to the organic form (complexes with proteins) and its distribution to the other tissues ^44^. This was further confirmed by the increase of Hg_F2_ concentration in muscle strips and whole tunicates along with the increase of Hg_F1_ concentration in stomachs and intestines of *S. thompsoni* (r = 0.90, *p* < 0.05) (Table 2).

**Table 2:** Correlation coefficients of total mercury and its fractions concentrations in whole organisms; stomachs, and intestines; and muscle strips and tunics of *Salpa thompsoni* (coefficients marked in bold were statistically significant, *p<0.05*), in 2018.

	THg	Hg_F1_	Hg_F2_	Hg_F3_	Hg_F4_	Hg_F5_
Whole organisms						
THg	1.00	**0.88**	**0.93**	− 0.14	− 0.53	0.00
Hg_F1_	**0.88**	1.00	**0.95**	0.18	− 0.70	− 0.20
Hg_F2_	**0.93**	**0.95**	1.00	0.13	− 0.58	− 0.32
Hg_F3_	− 0.14	0.18	0.13	1.00	0.25	− 0.24
Hg_F4_	− 0.53	− 0.70	− 0.58	0.25	1.00	0.30
Hg_F5_	0.00	− 0.20	− 0.32	− 0.24	0.30	1.00
Stomachs and intestines						
THg	1.00	0.18	0.69	− 0.27	− 0.42	− 0.34
Hg_F1_	0.18	1.00	0.35	0.03	− **0.74**	− 0.33
Hg_F2_	0.69	0.35	1.00	0.05	− **0.74**	− 0.24
Hg_F3_	− 0.27	0.03	0.05	1.00	− 0.17	**0.71**
Hg_F4_	− 0.42	− **0.74**	− **0.74**	− 0.17	1.00	0.16
Hg_F5_	− 0.34	− 0.33	− 0.24	**0.71**	0.16	1.00
Muscle strips and tunics						
THg	1.00	**0.89**	**0.98**	0.19	− 0.12	0.14
Hg_F1_	**0.89**	1.00	**0.90**	− 0.03	− 0.53	− 0.03
Hg_F2_	**0.98**	**0.90**	1.00	0.31	− 0.13	0.30
Hg_F3_	0.19	− 0.03	0.31	1.00	0.64	**0.96**
Hg_F4_	− 0.12	− 0.53	− 0.13	0.64	1.00	0.56
Hg_F5_	0.14	− 0.03	0.30	**0.96**	0.56	1.00

Significant values are in bold.

### Percentage fraction of Hg in THg

In the case of Antarctic salps, the Hg_F2_ fraction had the highest mean percentage (65%) and the Hg_F5_ fraction the lowest (0.2%). Comparable values were obtained during studies on selected benthic fauna from the Baltic Sea region, e.g., the Baltic mussels (*Limecola balthica*) or the crabs (*Eriocheir sinensis*) ^45^. The highest amount of fraction Hg_F1_, i.e., water-soluble compounds e.g., HgCl_2_, HgBr_2_, HgI_2_ was determined in muscle belts and tunics of salps (21%). In the literature, the highest proportion of Hg_F1_ in THg was obtained in phytoplankton (ca. 50–70%) ^45,46^. A study conducted by Bełdowska et al. ^46^ showed that Hg is adsorbed on the cells of marine phytoplankton mainly in the form of Hg chloride, bromide, and iodide, i.e., compounds included in the labile fraction Hg_F1_^46^. The concentration of organic forms of Hg (Hg_F2_), characterized by a high bioavailability, showed the highest proportion (71%) in the stomachs and intestines of salps, which was probably related to the intensive absorption of this fraction from the filtered particles. The Hg_F4_ fraction had a higher proportion in muscle strips and tunics (13%) than in stomachs and intestines (9%), which have been discussed in the section of *THg and Hg fractions in different body parts of Salpa thompsoni*. The percentage of Hg_F4_ in THg in muscle strips and tunics of Antarctic salps increased with decreasing Hg_F1_ (r = − 0.93, *p* < 0.05), as well as for the whole organisms in which the same trend has been observed with decreasing Hg_F2_ (r = − 0.88, *p* < 0.05) (Table 3). Sulfur compounds of Hg have a higher affinity for accumulation in organic matter than halide-bound Hg ^41^. This may be also related to the adsorption on a body surface of HgSO_4_ which may originate from the volcanic activity. About 250 km from the study area there is an active volcano Deception Island ^47^, which is one of the most active in the Southern Ocean, with more than 20 eruptions recorded in the past two centuries ^48^. Various forms of Hg, including HgSO_4,_ are emitted during eruptions or submarine fumes. For example, the influence of volcanism on increased mercury concentrations in lichens occurring on Deception Island area was demonstrated by Mão de Ferro et al. ^49^. Following eruptions in the 1960s and 1970s, newly formed bottomward density currents were observed in various areas of Deception Island, and these moves have significantly contributed to the remobilization of Hg from sediments ^48^. Additionally, increased geothermal activity from the volcano is thawing permafrost, which can be also a secondary source of Hg to the marine environment ^48^.

**Table 3:** Correlation coefficients of percentages (%) of mercury fractions in Hgtot in different parts of *Salpa thompsoni* (coefficients marked in bold were statistically significant, *p* < 0.05), in 2018.

	Hg_F1_	Hg_F2_	Hg_F3_	Hg_F4_	Hg_F5_
Whole organisms (%)					
Hg_F1_	1.00	0.65	− 0.66	− **0.93**	− 0.62
Hg_F2_	0.65	1.00	− 0.64	− **0.88**	− 0.55
Hg_F3_	− 0.66	− 0.64	1.00	0.69	0.62
Hg_F4_	− **0.93**	− **0.88**	0.69	1.00	0.63
Hg_F5_	− 0.62	− 0.55	0.62	0.63	1.00
Stomachs and intestines (%)					
Hg_F1_	1.00	− 0.09	− 0.10	− 0.67	− 0.34
Hg_F2_	− 0.09	1.00	− 0.14	− 0.68	− 0.24
Hg_F3_	− 0.10	− 0.14	1.00	0.13	**0.79**
Hg_F4_	− 0.67	− 0.68	0.13	1.00	0.38
Hg_F5_	− 0.34	− 0.24	**0.79**	0.38	1.00
Muscle strips and tunics (%)					
Hg_F1_	1.00	0.57	− 0.55	− **0.93**	− 0.30
Hg_F2_	0.57	1.00	− 0.06	− 0.81	0.33
Hg_F3_	− 0.55	− 0.06	1.00	0.30	0.77
Hg_F4_	− **0.93**	− 0.81	0.30	1.00	0.00
Hg_F5_	− 0.30	0.33	0.77	0.00	1.00

Significant values are in bold.

### Changes in Hg concentration versus size of organisms

The determined concentrations of total Hg in whole *S. thompsoni* organisms were in a relatively wide range (Table 1), which could be related due to the size of individuals, i.e., most likely their stage of life. The highest THg concentrations (mean 50.6 ng/g dw) were found in samples with individuals of the smallest size (0.1–1 cm), while lower and lowest values were characteristic for animals from the group of 1.1–3 cm size (mean 23.5 ng/g dw). The largest individuals (3.1–6 cm) appeared only in a one sample (n = 5), and the THg concentration (27.6 ng/g dw) was higher than in salps from 1.1 to 3 cm group. The results presented above suggest that in larger representatives of *S. thompsoni* Hg concentration is diluted with the growth of individuals—organism growth faster than bioaccumulation of the toxic element. Consequently, small organisms were characterized by the highest Hg concentrations. Analogous processes were previously observed by other authors in the Antarctic krill ^22^. Hg fractions, Hg_F1_, Hg_F2_, Hg_F3_ and Hg_F5_ showed the higher concentrations in smaller individuals. Only Hg_F4_ concentrations were higher in medium-sized organisms, which may be related (besides its transfer from different body compartments) to the intense of adsorption of HgSO_4_.

### Effects of local sources on Hg concentrations in *Salpa thompsoni*

**Figure 1:**
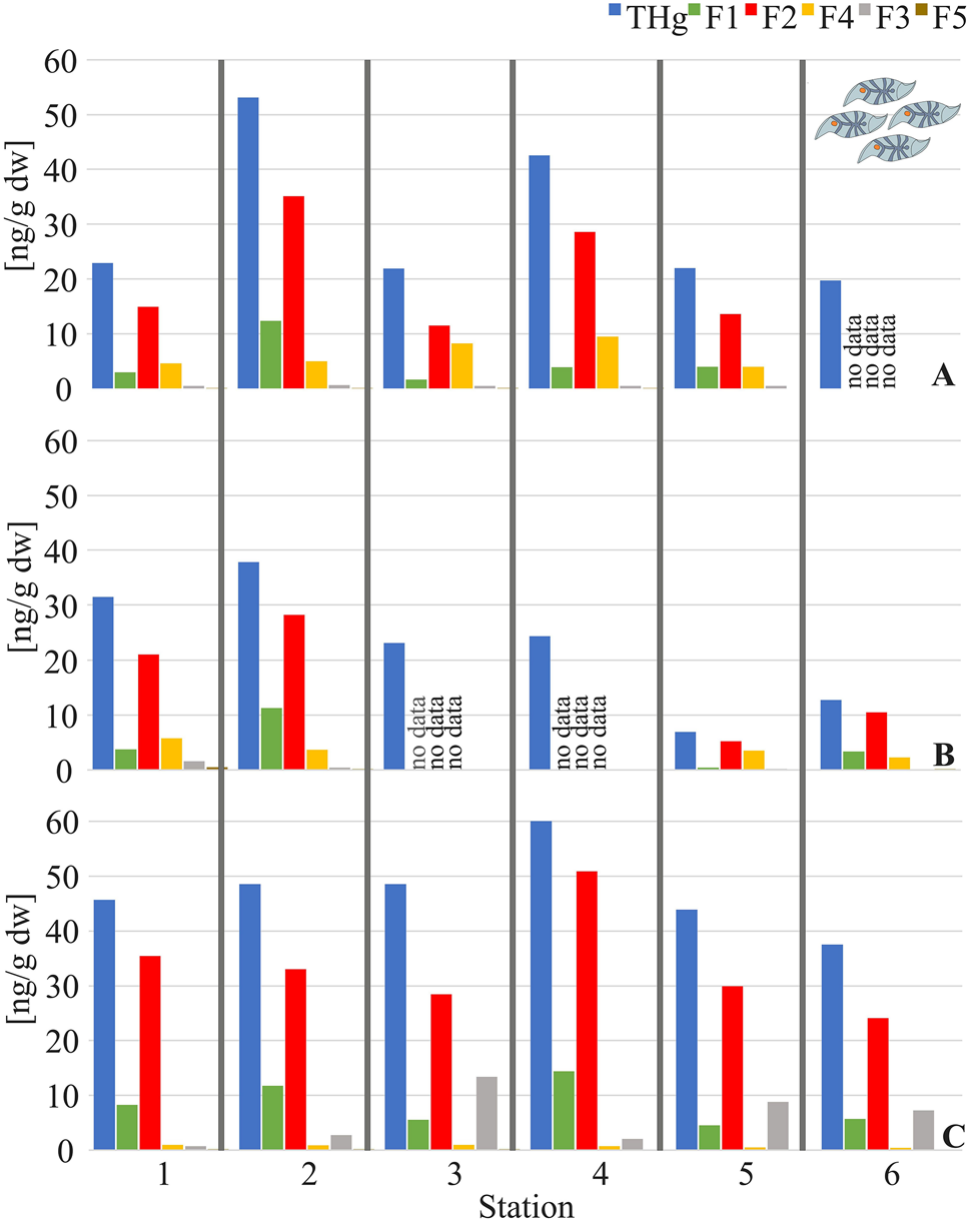
Concentrations of total mercury and its fractions in *Salpa thompsoni* individuals at each station (**A**) in whole organisms, (**B**) in muscle strips and tunics, (**C**) in stomachs and intestines (stations in order from closest to land to farthest away), in 2018.

**Figure 2:**
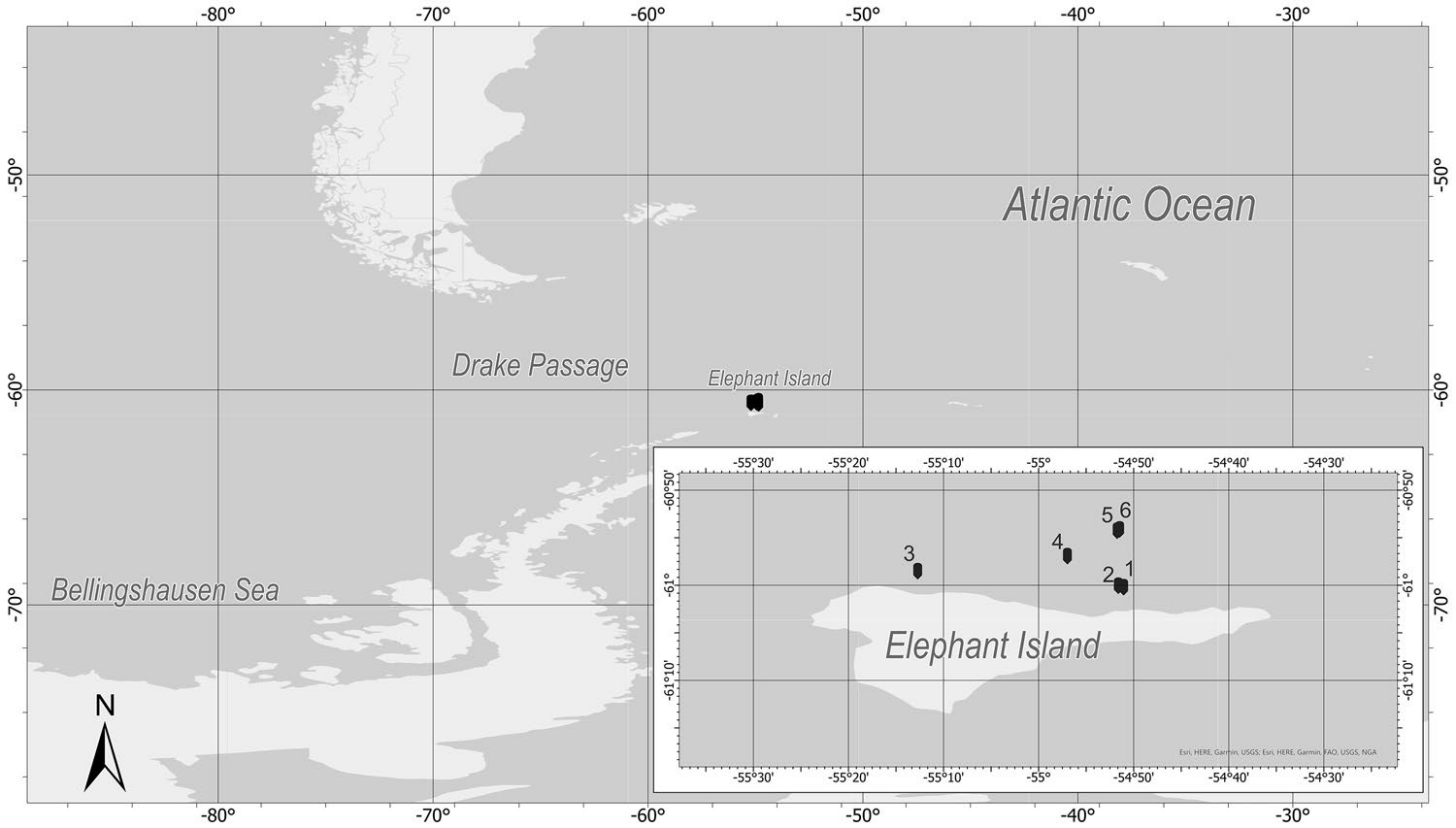
Location of research stations in the waters near Elephant Island (Antarctic Peninsula, Antarctica), in 2018.

The highest concentrations of THg, Hg_F1_, and Hg_F2_ were determined at station 2 and 4 (Fig. 1), which was located near Elephant Island (Fig. 2). In contrast, almost three times lower values were recorded at the station furthest from its coast. Considering these observations, it can be concluded that the most important factor influencing the Hg concentration in this area was probably the numerous concentrations of predators such as pinnipeds and sea birds (mainly penguins), which are stationed on Elephant Island. Hg accumulates in marine organisms by a process of bioaccumulation and biomagnification, mainly through the oral route. Hg concentrations in mammals and birds can be tens of thousands of times higher than in ambient waters ^50^. The chemical element has a high affinity for sulfhydryl groups, i.e., for most proteins, which makes it embedded, for example, in muscles, in fur, feathers, claws or in the eggshell, in the case of birds ^51,52^. This is a kind of detoxification process of the body, because during molting or breeding seasons, mammals or birds get rid of a certain amount of Hg from the body to the surrounding environment, where it can be reincorporated ^53,54^. Additionally, animals eliminate a relatively large load of Hg with their feces ^27,55,56^. This can be a significant source of Hg in the environment, especially in areas which are free of anthropogenic sources ^57^. However, these sources have more of a local rather than a regional impact. High concentrations of fractions containing HgO, HgSO_4_, Hg_F2_, in whole organisms among others, were determined at stations 3 and 4 (Fig. 1). Previous studies indicated that sediments and seawater surrounding this island contain elevated concentrations of some trace metals, including Hg, as well as elements such as sulfur, fluorine, and chlorine, which may be the result of volcanic processes in the South Shetland Islands area ^49,58^.

## Conclusions

This study presents the first results of THg and its fractions in one of the most ecologically important Antarctic zooplankton species *S. thompsoni*. along with fragmentation into organs. With the Hg fraction results, it can be concluded that the main route of exposure of *S. thompsoni* to Hg is presumably absorption from the food (filtered organic and non-organic particles). The transformation and embedding of simple, soluble Hg compounds into organic tissue, probably occurs in the stomachs and intestines. Higher THg concentrations, determined in small salps rather than in medium size individuals may be a result of the surface area to volume ratio, and a faster growth process than bioaccumulation of the investigated neurotoxin—bio-dilution. The main observed sources affecting Hg concentrations and Hg_F1_ (mainly Hg bound to halides) and Hg_F2_ (mainly organic Hg and bound to organic matter) fractions in *S. thompsoni* at the station closest to land were probably the detoxification process of mammals and seabirds on Elephant Island. On the other hand, an important source of THg and the Hg_F4_ fraction (manly HgSO_4_) adsorbed on the surface of the gelatinous body could be due to the past or current volcanic activity in the region. The source of the stable Hg fraction in *S. thompsoni* was presumably mineral suspension transported by meltwater from glaciers. Given that salps are non-selective filterers, monitoring of the stable fraction may be proper bioindicator of the amount of mineral suspension released from melting glaciers.

Salps can produce large quantities of dense, fast-sinking fecal pellets, and this makes them to be considered as an important transporter of particulate matter to sediments and bottom waters ^19^. Given that the highest THg concentrations were determined in stomachs and intestines, it seems possible that Hg which is transported with the pellets can be a source of the toxic element to the Southern Ocean sediments. However, for a better understanding of the sources of mercury in the Antarctic fauna, more detailed studies relating to the composition of organic and inorganic particles present in the water column, as well as detailed studies of the food content of the analyzed animals, would be necessary.

## Materials and methods

### Sampling methods and study area

The research material was collected in the West Antarctic Peninsula (WAP) region, from an area in close distance to the Elephant Island (research expedition PS112, Project KRILLBIS (Krill Biomass Estimation in the Southern Ocean), 17.03–06.05.2018)). Salps were collected from 6 stations (Fig. 2, Table 4), using an IKMT-type plankton net with an inlet section area of 1.8 m^2^ and a mesh size diameter of 505 µm. The net was towed diagonally to a maximum depth of 170 m, the deepest 20 m from the bottom, at a maximum speed of 1–2 knots. Only blastozooids were collected for this study due to their much higher abundance in samples. The number of organisms ranged from 10 to 478 individuals per station (775 in total). After collection, *S. thompsoni* samples were stored at − 20 °C. Each sample was thawed and rinsed with distilled water prior to chemical analyses. Organisms were divided into three size classes: 0.1–1 cm; 1.1–3 cm; 3.1–6 cm. Salps were examined as whole organisms, or internal organs (stomachs and intestines) and muscle strips and tunics were extracted. All tissues were lyophilized (Alpha 1–4 LDplus, Martin Christ, Germany) and then homogenized in an agate mortar.

**Table 4:** Coordinates of research stations in the area of Elephant Island, in 2018.

Station	Latitude	Longitude
1	− 61.01806	− 54.85097
2	− 61.01555	− 54.86027
3	− 60.99004	− 55.21196
4	− 60.96362	− 54.94955
5	− 60.91912	− 54.86233
6	− 60.91687	− 54.85795

Elephant Island is part of the South Shetland Islands, which are in the Western Antarctic Peninsula region (WAP). WAP extends north from the northwestern part of the Antarctic continent toward the southern tip of South America. Elephant Island is one of the northernmost Antarctic island in this area. In recent decades, there has been observed a notable decrease in the ice cover and extent of the ice shelves of the WAP region because of climate change ^13,25^. Moreover, in the investigated area there are many research stations, krill fishery is active and tourist interest is growing. Additionally, not far from Elephant Island (about 250 km) is the active volcano Deception Island ^48^.

### Hg analysis

Determination of THg and its five fractions were performed using a direct mercury analysis DMA-80 (Milestone, Italy). The analysis of THg consisted of thermal decomposition of the sample at 750 °C, amalgamation of Hg vapors and detection of atomic absorption of Hg, using oxygen as a carrier gas ^59^. For analysis, approximately 60 mg of sample was weighed into quartz boats each, then placed in a DMA—80 autosampler. The correctness of the analysis was verified by the analysis of certified reference materials (tea leaves INCTTL-1—THg: 5 ng/g, soil NCS DC 87,103–THg: 17 ng/g, plankton BCR-414–THg: 276 ng/g), at mean recovery > 95% and relative standard deviation (RSD) > 85%. The limit of detection (LOD) was calculated based on SD of Hg concentration in the blank samples, measured in 10 repetitions (LOD = 3 × SD). In the case of analysis of THg, LOD was 1 pg Hg. More details were described by Wilman et al. ^60^. The analysis of Hg fractions was based on a 5-step method developed by Bełdowska et al. ^46^ and modified by Wilman et al. ^60^. This analysis allows to separate five groups of compounds with similar properties. Its course is analogous to that of THg, but the sample is burned sequentially at different temperatures. The first fraction (Hg_F1_) is released at 175 °C and these are the most unstable, water-soluble Hg compounds—mainly halogens: HgCl_2_, HgBr_2_, HgI_2_, Hg (CN)_2_. In the next step, at 225 °C, mainly Hg associated with organic matter and organic forms of Hg (including methylmercury) (Hg_F2_) are released. These Hg compounds are much more easily absorbed into the body than inorganic salts. At 325 °C, the most stable and least toxic form of Hg, HgS (Hg_F3_), is released. In the next temperature of 475 °C, unstable compounds (Hg_F4_) such as HgO, HgSO_4_, HgF_2_ are decomposed. At the highest temperature of 750 °C, Hg is decomposed in its least available form attached to minerals—residual Hg (Hg_F5_) ^60^. Five fractions were determined based on the internal reference material. The quality control of analyzes were done at least once per week. There are no reference materials for Hg speciation (except for MeHg). Therefore, the method was tested on certified reference materials (tea leaves – INCT-TL-1; soil – NCS DC 87,103), on the 3 synthetic (HgCl_2_, HgS, HgO) (Sigma Aldrich Co., purity ≥ 97%), as well as on natural materials (seal muscle). Natural samples designated for testing the method, before chemical analyses, were previously lyophilized and homogenized in a ball mill, so that the sample was as homogeneous as possible. Then, the sample was stored in a cabinet desiccator under constant conditions of temperature and humidity until analysis. The Hg standards were chosen for examination: HgCl_2_, HgS, HgO were characterized by a high Hg concentration (which was too high to be measured directly). Samples were therefore dry diluted by mixing with a substrate, namely beach sand (> 90% SiO_2_), previously heated for 24 h at 800 °C as was described by Wilman et al. ^60^. The analysis was verified by comparing the sum of Hg concentrations measured by means of the 5-stage fractionation method with the result of the analysis of THg concentration. Mean recovery from the analysis of forms of Hg was 101%, with a standard deviation not exceeding 5%. LOD for each fractionation method was calculated based on ten repetitions of substrate analysis. The calculated LOD values were at a level of 1 pg Hg, like in the case of THg. More details of the methodology were described by Wilman et al. ^59^.

Statistical analysis of the obtained results were performed using *STATISTICA* 12. Pearson correlations were calculated, and results with p < 0.05 were considered as a statistically significant.

## Data Availability

The datasets used and/or analysed during the current study available from the corresponding author on reasonable request.
